# Beverage-Derived Caffeine Intake, Sleep Duration, and Mental Health Among Korean Adults: A Cross-Sectional Analysis of KNHANES 2021–2024

**DOI:** 10.3390/nu18152488

**Published:** 2026-08-01

**Authors:** Jinmi Kim, Yu Hyeon Yi

**Affiliations:** 1 Department of Biostatistics, Clinical Trial Center, Biomedical Research Institute, Pusan National University Hospital, Busan 49241, Republic of Korea; jinmi@pusan.ac.kr; 2 Department of Family Medicine and Medical Research Institute, Pusan National University Hospital, Busan 49241, Republic of Korea; 3 Department of Family Medicine, Pusan National University School of Medicine, Yangsan 50612, Republic of Korea

**Keywords:** caffeine intake, anxiety, mental health, Korea National Health and Nutrition Examination Survey (KNHANES)

## Abstract

**Background/Objectives:** To examine the association between caffeine intake and mental health- and sleep-related outcomes, including anxiety, sleep duration, and perceived stress, in a nationally representative sample of Korean adults. **Methods:** We analyzed 20,504 participants from the Korea National Health and Nutrition Examination Survey (KNHANES) 2021–2024. Caffeine intake was categorized into six groups, and mental health- and sleep-related outcomes included clinically significant anxiety symptoms (GAD-7 ≥ 10), sleep duration, and perceived stress. Survey-weighted multivariable logistic regression models and restricted cubic spline analyses were used to evaluate associations between caffeine intake and mental health- and sleep-related outcomes, adjusting for demographic, socioeconomic, behavioral, dietary, and clinical factors. **Results:** The prevalence of clinically significant anxiety symptoms was 4.7%. Restricted cubic spline analyses demonstrated a non-linear association between caffeine intake and anxiety (*p*-overall = 0.010; *p*-nonlinear = 0.016). Higher caffeine intake was associated with progressively increasing odds of short sleep duration (<6 h/day), indicating a clear dose–response relationship (*p*-overall < 0.001; *p*-nonlinear < 0.001). Higher caffeine intake was also associated with increased perceived stress (*p*-overall = 0.025), although evidence for a nonlinear relationship was weaker (*p*-nonlinear = 0.062). These findings were generally consistent across multivariable-adjusted models. **Conclusions:** Caffeine intake is differentially associated with mental health- and sleep-related outcomes, with excessive intake related to adverse effects on sleep and stress.

## 1. Introduction

Caffeine is one of the most widely consumed psychoactive substances worldwide, commonly ingested through coffee, tea, and energy drinks [1,2]. Its stimulating effects on the central nervous system are primarily mediated through non-selective antagonism of adenosine A1 and A2A receptors, leading to increased neuronal activity and indirect modulation of dopaminergic neurotransmission. As a result, caffeine enhances alertness, reduces fatigue, improves attention, concentration, and mood, and promotes a sense of well-being [3,4,5]. In addition, habitual caffeine consumption has been associated with potential long-term neuroprotective effects, including a lower risk of cognitive decline and neurodegenerative diseases such as Parkinson’s disease and Alzheimer’s disease [6,7]. However, caffeine also influences sleep regulation and emotional states, raising concerns about its potential impact on mental health, particularly at higher levels of intake [3,8].

Previous studies have reported inconsistent findings regarding the relationship between caffeine intake and mental health and sleep [9]. While moderate consumption has been associated with beneficial or neutral effects, excessive intake may exacerbate anxiety symptoms through increased physiological arousal and sleep disruption. In addition, individuals with anxiety may intentionally reduce caffeine intake, complicating the directionality of this association [10]. As a result, the overall relationship may not be linear. Sleep disturbance is closely associated with both caffeine intake and mental health outcomes [5]. Caffeine acts as an adenosine receptor antagonist, delaying sleep onset and reducing sleep duration and quality [11]. In addition, insufficient sleep has been consistently associated with adverse mental health outcomes, including anxiety and perceived stress [12,13].

Despite these considerations, important knowledge gaps remain. Most previous epidemiological studies have focused on a single mental health outcome, have relied on simple measures of coffee consumption rather than quantitative caffeine intake, or have assumed linear dose–response relationships. In addition, population-based evidence from Asian populations remains limited despite substantial differences in beverage consumption patterns and caffeine sources compared with Western countries [14,15,16]. Few studies have simultaneously examined anxiety, sleep duration, and perceived stress using quantified caffeine intake while evaluating potential non-linear dose–response relationships and subgroup differences by sex and age [17,18].

Therefore, this study aimed to examine the association between beverage-derived caffeine intake and multiple mental health- and sleep-related outcomes, including clinically significant anxiety symptoms, short sleep duration, and perceived stress, using nationally representative data from Korean adults participating in KNHANES 2021–2024. We further evaluated potential non-linear dose–response relationships to better inform public health recommendations on caffeine consumption.

## 2. Materials and Methods

### 2.1. Study Population

**Figure 1:**
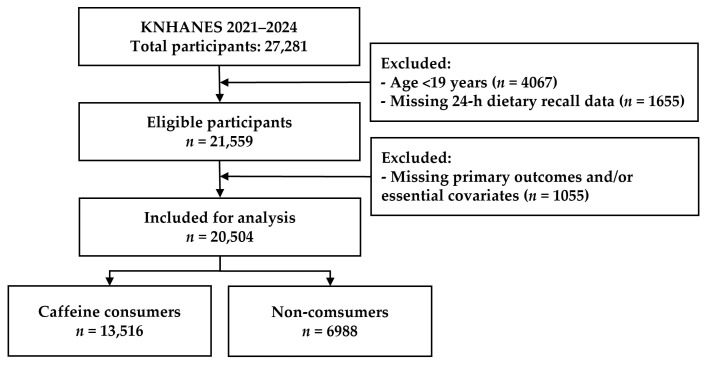
Flowchart of study population selection and classification according to caffeine consumption in KNHANES 2021–2024. “Non-consumers” refers to participants with 0 mg/day caffeine intake on the 24 h dietary recall day and should not be interpreted as habitual caffeine abstainers.

Data were obtained from the Korea National Health and Nutrition Examination Survey (KNHANES) conducted between 2021 and 2024, a nationally representative survey employing a complex, multistage probability sampling design. Participant selection is summarized in Figure 1. Of the initial 27,281 participants, we excluded those aged <19 years (*n* = 4067), those with missing 24 h dietary recall data (*n* = 1655), and those with missing data on the primary outcomes (GAD-7 score, perceived stress, or sleep duration) or essential covariates (smoking status, diabetes mellitus, or dyslipidemia) (*n* = 1055). Consequently, a total of 20,504 participants were included in the final analysis. Participants were classified according to beverage-derived caffeine intake estimated from the 24 h dietary recall data. Individuals who consumed caffeinated beverages were classified as caffeine consumers (*n* = 13,516), whereas those who consumed only caffeine-free beverages or reported no beverage consumption were classified as non-consumers (*n* = 6988).

### 2.2. Data Collection

The National Health and Nutrition Survey comprised a physical examination, health survey, and nutrition survey and obtained various information via self-administered questionnaires and interviews with investigators. Participants’ age, education level (elementary school or lower, middle school, high school, college or higher), household income (income quartile), marital status, smoking status (never, current, past), alcohol consumption (≤1 a month, 2–4 times a month, 2–3 times a week, ≥4 times a week), the frequency of morning meals (5–7 times a week, 3–4 times a week, 1–2 times a week, and 0 times a week), comorbidities (hypertension, dyslipidemia, diabetes), average daily sleep duration were obtained via a questionnaire. Total energy, carbohydrate, protein, fat, sodium, total dietary fiber, and total sugar were calculated through a 24 h food intake survey. Body mass index (BMI) was calculated based on measured height and weight.

The degree of anxiety was assessed using the Generalized Anxiety Disorder-7 (GAD-7), a self-report measurement tool [19]. Perceived stress was evaluated using a self-reported measure of usual stress levels. Responses were categorized into four levels ranging from very high to very low stress. Participants reporting very high or high stress were classified as having elevated perceived stress, whereas those reporting low or very low stress were classified as having lower perceived stress. Depressive symptoms were assessed by asking participants whether they had experienced feelings of sadness or hopelessness lasting for at least two consecutive weeks during the previous year. Suicidal ideation was assessed using the question, “Have you seriously considered suicide during the past 12 months?” Mental health counseling was assessed by asking participants whether they had received counseling for mental health concerns during the previous year.

### 2.3. Identification of Caffeinated Beverages

Dietary intake information was obtained from the 24 h dietary recall component of KNHANES. Beverage records were selected from food items classified within the beverage-related categories, including beverages and tea/coffee products. Because discrepancies were observed between standardized food codes and the actual beverage names reported by participants, beverage classification was based primarily on text information rather than food codes alone. To improve identification accuracy, a composite text field was created by combining the reported food name, detailed food description, and product name. Caffeinated beverages were then identified using a rule-based classification framework that incorporated predefined keyword dictionaries and regular-expression matching. Candidate beverages were first detected through text searches of the composite field. Subsequently, all records associated with the same beverage code were reviewed to capture products with inconsistent or incomplete labeling. Beverage-derived caffeine intake was estimated using the single 24 h dietary recall collected in KNHANES. Because KNHANES does not obtain repeated 24 h dietary recalls from the same participants, usual caffeine intake could not be estimated. Therefore, the calculated caffeine intake reflects caffeine consumption on the recall day rather than habitual intake. To enhance transparency and reproducibility, the beverage classification criteria, assigned caffeine concentrations, and corresponding KNHANES dish codes used for caffeine estimation are provided in Supplementary Table S1.

### 2.4. Classification of Beverage Subtypes

Caffeinated beverages were grouped into five categories: coffee, tea (including green tea and other tea products), energy drinks, cola-type beverages, and cocoa-based beverages [20].

Coffee beverages were subdivided into liquid coffee, instant coffee powder, prepared coffee mixes, and roasted bean or capsule coffee. Tea beverages were classified as liquid, powdered, or infusion-type. Energy drinks, cola-type beverages, and cocoa-based beverages were treated as single subtypes [20]. Decaffeinated beverages were identified using dedicated keywords.

### 2.5. Assignment of Caffeine Content and Estimation of Daily Intake

Caffeine concentrations (mg/L) were assigned using mean values from national monitoring data published by the Ministry of Food and Drug Safety [20]. The applied mean caffeine concentrations were: liquid coffee (352.9 mg/L), instant coffee powder (293.5 mg/L), prepared coffee mixes (571.9 mg/L), roasted bean or capsule coffee (522.8 mg/L); liquid tea (105.8 mg/L), powdered tea (115.0 mg/L), infusion-type tea (143.6 mg/L); energy drinks (320.8 mg/L); cola beverages (187.5 mg/L); and cocoa-based beverages (360.9 mg/L). Decaffeinated beverages were assigned a nominal caffeine concentration of 1 mg/L to reflect minimal residual caffeine content.

In the KNHANES 24 h dietary recall, the intake amount of each reported beverage item is recorded in either volume (mL) or weight (g), depending on the standard unit defined for the corresponding food code. For items recorded in grams—mainly powder- or capsule-based products such as instant coffee, coffee mixes, and powdered tea—intake was treated as numerically equivalent in milliliters (1 g ≈ 1 mL) and combined with the volume (mL)-recorded items to derive total beverage volume (L).

Caffeine intake per record (mg) was calculated by multiplying beverage volume (L) by the assigned concentration (mg/L). Total daily caffeine intake (mg/day) was obtained by summing intake across all beverages consumed on the recall day.

### 2.6. Statistical Analysis

Statistical analyses accounted for the complex sampling design of KNHANES by incorporating sampling weights, stratification variables, and primary sampling units. Combined sampling weights for the health interview, health examination, and nutrition survey were used. For the pooled 2021–2024 data, the annual sampling weights were divided by four in accordance with the KNHANES analytic guidelines for combining multiple survey cycles to obtain nationally representative estimates [21]. Participant characteristics were summarized as unweighted frequencies with weighted percentages for categorical variables and weighted means ± standard errors (SEs) for continuous variables. Differences across caffeine intake categories were assessed using design-based Pearson’s chi-square tests with Rao–Scott adjustment for categorical variables and design-based Kruskal–Wallis tests for continuous variables.

To evaluate the associations between caffeine intake and mental health- and sleep-related outcomes, including clinically significant anxiety symptoms (GAD-7 score ≥ 10) [22], short sleep duration (<6 h/day), and perceived stress, survey-weighted generalized linear models with a quasi-binomial distribution were fitted. Short sleep duration was defined as <6 h/day according to previous epidemiologic studies and sleep health [23]. Restricted cubic spline models were used to evaluate potential nonlinear associations between caffeine intake and each outcome. Four knots were placed at the 5th, 35th, 65th, and 95th percentiles of caffeine intake. A caffeine intake of 100 mg/day was used as the reference value to maintain consistency with the reference category (100 to <200 mg/day) used in the categorical analyses. Predicted odds ratios were generated from 0 mg/day to the 99th percentile of the observed caffeine intake for graphical presentation. Overall and nonlinear associations were assessed using Wald tests within the survey-weighted regression models. Covariates included in the multivariable models were selected a priori based on previous epidemiological literature and investigator consensus regarding their potential role as confounders of the association between caffeine intake and mental health outcomes. Models were adjusted for potential confounding factors, including demographic characteristics (sex and age group), socioeconomic status (education level, household income quartile, marital status, and body mass index [BMI]), health-related behaviors (alcohol consumption and smoking status), dietary factors (breakfast frequency, total energy intake, carbohydrate, protein, fat, sodium, dietary fiber, and total sugar intake), and clinical comorbidities (hypertension, diabetes mellitus, and dyslipidemia). Adjusted odds ratios (aORs) and 95% confidence intervals (CIs) were calculated.

All statistical tests were two-tailed, and a *p* value < 0.05 was considered statistically significant. Statistical analyses were performed using R (version 4.4.2; R Foundation for Statistical Computing, Vienna, Austria) with the survey and splines packages.

### 2.7. Missing Data

Participants with missing primary outcomes or essential covariates (smoking status, diabetes mellitus, or dyslipidemia) were excluded from the analyses, as described above. Missing values for the remaining covariates included in the multivariable models were retained as separate categories in the primary analyses to maximize sample retention and preserve the representativeness of the KNHANES complex survey data. To evaluate the robustness of this missing-data handling strategy, we additionally performed a complete-case sensitivity analysis excluding participants with missing values for any remaining covariates included in the fully adjusted model (Sensitivity Analysis V).

### 2.8. Sensitivity Analysis

Five sensitivity analyses were performed to evaluate the robustness of the main findings. First, all cocoa beverages were assigned a caffeine concentration of 0 mg/L (Sensitivity Analysis I). Second, decaffeinated beverages were additionally assigned a caffeine concentration of 0 mg/L (Sensitivity Analysis II). Third, pregnant women and participants with implausible total energy intake (<500 or ≥5000 kcal/day) were excluded (Sensitivity Analysis III). Fourth, the fully adjusted model was re-fitted after excluding dietary covariates (breakfast frequency, total energy intake, carbohydrate, protein, fat, sodium, dietary fiber, and total sugar intake) to evaluate the influence of dietary adjustment (Sensitivity Analysis IV). Finally, a complete-case analysis excluding participants with missing values for any covariates included in the fully adjusted model was performed (Sensitivity Analysis V). Sensitivity analyses were performed cumulatively, with each subsequent analysis incorporating the modifications introduced in the preceding analyses (Supplementary Table S2).

## 3. Results

A total of 20,504 participants from KNHANES 2021–2024 were included in the analysis, of whom 66.7% were caffeine consumers. The prevalence of clinically significant anxiety symptoms (GAD-7 score ≥ 10) was 4.7%.

Baseline characteristics according to caffeine consumption are presented in Table 1. Compared with non-consumers, caffeine consumers were more likely to be male and middle-aged (45–64 years), whereas non-consumers were more likely to be older adults (≥65 years) (*p* < 0.001). Caffeine consumers had higher socioeconomic status, with greater proportions of college education and higher household income (*p* < 0.001). They were also more likely to be married. In terms of health-related behavior, caffeine consumers reported more frequent alcohol consumption and were more likely to be current or past smokers (*p* < 0.001). Dietary intake was consistently higher among caffeine consumers, including total energy, macronutrients, sodium, and sugar intake (all *p* < 0.001). Regarding comorbidities, hypertension was slightly less prevalent among caffeine consumers, whereas diabetes and dyslipidemia did not differ significantly between groups.

The distribution of beverage-derived caffeine intake is presented in Supplementary Table S3. The weighted median intake was 75.8 mg/day, with substantial right-skewness (P25–P75: 0–185.6 mg/day; P99: 622.1 mg/day). Mental health outcomes varied across levels of caffeine intake (Table 2). Mean GAD-7 scores was associated with higher caffeine intake, reaching the highest levels among those consuming 300–400 mg/day. The prevalence of clinically significant anxiety symptoms (GAD-7 score ≥ 10) showed a non-linear pattern, with lower prevalence at moderate intake levels (<200 mg/day) and higher prevalence at ≥300 mg/day (*p* < 0.001). Higher caffeine intake was also associated with shorter sleep duration, with the highest proportion of sleep <6 h observed in the ≥400 mg/day group (16.7%) (*p* < 0.001). Similarly, perceived stress, depression, and mental health counseling were more common at higher levels of caffeine intake, particularly ≥300 mg/day. Suicidal ideation showed a modest but statistically significant variation across groups.

**Figure 2:**
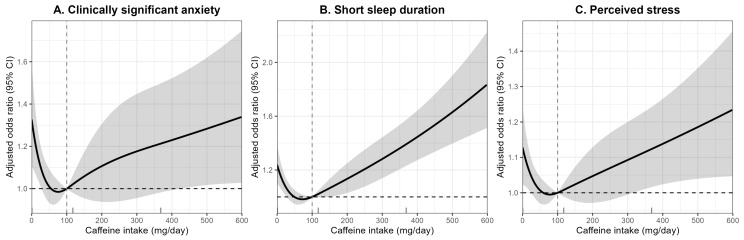
Restricted cubic spline associations between caffeine intake and mental health outcomes in the overall population. (**A**) Clinically significant anxiety symptoms (GAD-7 score ≥ 10); (**B**) short sleep duration (<6 h/day); and (**C**) perceived stress. Adjusted odds ratios (ORs; solid black line) and 95% confidence intervals (CIs; gray shaded area) were estimated from the fully adjusted model, with 100 mg/day as the reference value (vertical dashed line); the horizontal dashed line indicates an OR of 1. Short vertical marks along the *x*-axis indicate the locations of the restricted cubic spline knots at the 5th, 35th, 65th, and 95th percentiles of caffeine intake.

Restricted cubic spline analyses showed dose–response associations between caffeine intake and mental health outcomes in the overall population (Figure 2). Predicted odds ratios from the restricted cubic spline models are presented in Supplementary Table S4.

For clinically significant anxiety symptoms, a significant overall association (*p*-overall = 0.010) and evidence of nonlinearity (*p*-nonlinear = 0.016) were observed in the fully adjusted model. The lowest odds were observed at approximately 70–100 mg/day, whereas both lower and higher caffeine intake levels were associated with increased odds of anxiety.

For short sleep duration, caffeine intake was strongly associated with increasing odds of short sleep (*p*-overall < 0.001). The odds increased progressively with higher caffeine intake, although evidence of nonlinearity was also observed (*p*-nonlinear < 0.001).

For perceived stress, higher caffeine intake was associated with increased odds of perceived stress (*p*-overall = 0.025). However, evidence for nonlinearity was weaker and did not reach statistical significance in the fully adjusted model (*p*-nonlinear = 0.062). The observed pattern was generally characterized by increasing odds with higher caffeine intake.

Fully adjusted categorical associations are presented in Table 3, and categorical associations across sequential adjustment models are presented in Supplementary Table S5. No significant interaction by sex or age group was observed for clinically significant anxiety symptoms (*p*-interaction = 0.378 and 0.474, respectively), short sleep duration (*p*-interaction = 0.525 and 0.068, respectively), or perceived stress (*p*-interaction = 0.346 and 0.395, respectively).

## 4. Discussion

In this nationally representative study of Korean adults, caffeine intake showed a complex association with mental health- and sleep-related outcomes. While moderate intake was associated with lower prevalence of anxiety, both non-consumption and higher intake levels (≥300 mg/day) were linked to increased odds of clinically significant anxiety symptoms, suggesting a non-linear association. In contrast, higher caffeine intake demonstrated a clear dose–response association with short sleep duration and perceived stress.

Anxiety symptoms are influenced by multiple demographic, lifestyle, and health-related factors [24]. Previous studies have identified female sex, younger age, lower socioeconomic status, smoking, alcohol use, sleep disturbance, obesity, and chronic diseases as important determinants of anxiety symptoms [25,26,27]. In addition, sleep-related factors have consistently shown strong associations with anxiety, with short sleep duration and poor sleep quality contributing to emotional dysregulation and increased psychological distress [28]. Dietary factors have also gained attention, particularly caffeine intake, because caffeine acts as a central nervous system stimulant through antagonism of adenosine receptors and may increase sympathetic activity, arousal, and stress responses [29,30].

The present study extends previous evidence by demonstrating non-linear associations between beverage-derived caffeine intake and mental health- and sleep-related outcomes in a nationally representative Korean population using harmonized caffeine estimates across multiple KNHANES cycles. While previous studies mainly focused on total caffeine intake or single beverage sources in limited populations [17,31], our approach enabled standardized estimation of caffeine intake from diverse beverages. We observed that higher caffeine intake was associated with short sleep duration, perceived stress and with elevated odds of clinically relevant anxiety symptoms (GAD-7 score ≥ 10).

The observed non-linear association pattern for anxiety may reflect both behavioral and biological mechanisms [32]. Individuals with existing anxiety symptoms may avoid caffeine due to its known stimulatory effects [33], contributing to the elevated risk observed among non-consumers. Conversely, high caffeine intake may exacerbate anxiety through increased central nervous system stimulation, heightened arousal, and sleep disruption [10]. These findings are consistent with prior studies reporting bidirectional relationships between caffeine use and anxiety symptoms.

Caffeine is known to antagonize adenosine receptors, thereby delaying sleep onset and reducing sleep efficiency [5]. In this study, individuals consuming ≥ 400 mg/day had substantially higher odds of sleeping less than 6 h, with consistent findings across subgroups. Given the well-established link between insufficient sleep and adverse mental health outcomes, sleep disturbance may partially mediate the relationship between caffeine and anxiety [34]. Sleep duration was not included as an adjustment variable in the anxiety models because it could lie on the potential causal pathway between caffeine intake and anxiety. Instead, sleep duration and anxiety were analyzed as separate outcomes.

From a public health perspective, our findings are consistent with current recommendations to avoid excessive caffeine intake, particularly at levels ≥300–400 mg/day. In the present study, beverage-derived caffeine intake around 100 mg/day was associated with the lowest adjusted odds of clinically significant anxiety symptoms, whereas higher intake levels were associated with poorer sleep and greater perceived stress. However, these associations should be interpreted cautiously because of the cross-sectional design and the potential for residual confounding. Public health messaging should therefore emphasize moderation in caffeine consumption and consider its potential associations with sleep and mental well-being, particularly among individuals at risk for sleep disturbances or anxiety.

In addition, moderate caffeine consumption may reflect healthier lifestyle patterns or more favorable socioeconomic circumstances rather than the biological effects of caffeine itself. Although we adjusted for a broad range of demographic, socioeconomic, lifestyle, dietary, and clinical factors, residual confounding from unmeasured characteristics, such as health consciousness, occupational factors, daily routines, social functioning, and other health-promoting behaviors, cannot be excluded. Therefore, the observed associations should be interpreted with caution.

Several limitations should be acknowledged. First, because of the cross-sectional nature of this study, causal relationships cannot be established. Reverse causation remains a plausible explanation for the observed associations. Individuals experiencing anxiety, psychological distress, poor sleep, or other health conditions may intentionally reduce or avoid caffeine intake or, conversely, increase caffeine consumption to alleviate fatigue. Therefore, the observed non-linear association between caffeine intake and anxiety should not be interpreted as evidence that moderate caffeine intake protects against anxiety. Prospective longitudinal studies are needed to clarify the temporal relationship between caffeine intake and mental health- and sleep-related outcomes.

Second, caffeine intake was estimated from a single 24 h dietary recall and food composition data and therefore reflects caffeine intake on the recall day rather than habitual caffeine consumption. Accordingly, the observed associations should be interpreted as relationships between recall-day caffeine intake and self-reported longer-term outcomes, including two-week anxiety symptoms, habitual sleep duration, and perceived stress, rather than as evidence of habitual caffeine consumption. Because caffeine intake was assessed using a single 24 h dietary recall, participants classified as having no caffeine-containing beverage intake on the recall day should not be interpreted as habitual abstainers. This group is likely heterogeneous and may include occasional consumers, individuals temporarily avoiding caffeine-containing beverages, and those with caffeine exposure from sources not captured in the present analysis. In addition, caffeine content may vary according to beverage type, preparation method, serving size, and brand, which could not be fully accounted for in the present analysis. Although standardized caffeine concentrations were assigned to each beverage category, the actual caffeine content may vary according to preparation methods, product formulations, serving sizes, and coffee types, which could have resulted in some degree of exposure misclassification. Consequently, misclassification is possible and may have attenuated the observed associations. In addition, the relatively small number of anxiety cases in the ≥400 mg/day category (*n* = 40) may have limited the precision of the estimated associations in this subgroup. Because caffeine concentrations were assigned as category-level means rather than measured for individual products, some degree of non-differential exposure misclassification is likely. Such misclassification would generally be expected to bias associations toward the null, suggesting that the true associations between caffeine intake and the study outcomes may be at least as strong as those reported here. Sensitivity analyses in which the assigned caffeine concentrations for cocoa-based and decaffeinated beverages were set to 0 mg/L yielded materially consistent results (Supplementary Table S2), supporting the robustness of our findings to this source of measurement error.

Third, although we adjusted for a wide range of sociodemographic and lifestyle factors, residual confounding cannot be excluded. Important variables, including psychiatric history, psychotropic medication use, shift work, occupational stress, sleep timing, chronotype, and the timing of caffeine consumption, were not available in the KNHANES dataset and therefore could not be considered. These unmeasured factors may have influenced both caffeine intake and mental health- and sleep-related outcomes.

Fourth, perceived stress was assessed using a single self-reported question in KNHANES rather than a validated multi-item instrument such as the Perceived Stress Scale. Although this item has been widely used in national health surveys, it may not fully capture the multidimensional nature of psychological stress and may be subject to reporting bias or misclassification. Therefore, the observed associations with perceived stress should be interpreted cautiously.

Finally, this study evaluated total caffeine intake rather than specific caffeine-containing beverages. Coffee, tea, cola, energy drinks, and cocoa-based beverages differ not only in caffeine content but also in sugar content, bioactive compounds, consumption patterns, and timing of intake, all of which may differentially influence mental health- and sleep-related outcomes. Future studies examining beverage-specific associations may provide additional insight into these relationships.

Although cognitive function represents an important aspect of brain health, standardized cognitive assessments were not available in the KNHANES 2021–2024 dataset. Consequently, we were unable to examine the association between caffeine intake and cognitive performance. Future prospective studies incorporating validated cognitive measures are needed to clarify the relationship between caffeine consumption, cognition, and mental health.

## 5. Conclusions

In conclusion, caffeine intake was differentially associated with mental health- and sleep-related outcomes, showing a non-linear association with anxiety, short sleep duration and perceived stress. These findings should be interpreted as observational associations rather than evidence of causality. Longitudinal studies are needed to determine the direction and mechanisms underlying these relationships.

## Figures and Tables

**Table 1:** Baseline characteristics according to caffeine consumption.

Characteristic	Overall	Caffeine Consumers >0 mg/day	Non-Consumers 0 mg/day	*p* Value
	*N* = 20,504 (100%)	*N* = 13,516 (66.7%)	*N* = 6988 (33.3%)	
Demographics & anthropometrics				
Survey year				0.846
2021	4400 (24.3%)	2918 (24.3%)	1482 (24.2%)	
2022	4748 (25.0%)	3149 (25.2%)	1599 (24.6%)	
2023	5666 (25.4%)	3711 (25.2%)	1955 (25.8%)	
2024	5690 (25.4%)	3738 (25.3%)	1952 (25.4%)	
Sex				<0.001
Male	8848 (49.8%)	6093 (52.0%)	2755 (45.5%)	
Female	11,656 (50.2%)	7423 (48.0%)	4233 (54.5%)	
Age group (years)				<0.001
19–44	6508 (41.7%)	4152 (39.8%)	2356 (45.6%)	
45–64	7693 (37.1%)	5582 (41.0%)	2111 (29.2%)	
65+	6303 (21.2%)	3782 (19.2%)	2521 (25.2%)	
Body mass index (BMI, kg/m^2^)	24.18 ± 0.04	24.31 ± 0.04	23.91 ± 0.06	<0.001
Socioeconomic status				
Education				<0.001
≤Elementary school	3562 (11.6%)	2043 (10.1%)	1519 (14.6%)	
Middle school	2014 (7.6%)	1249 (7.2%)	765 (8.6%)	
High school	6754 (34.8%)	4453 (34.1%)	2301 (36.1%)	
≥College	8163 (45.9%)	5765 (48.6%)	2398 (40.7%)	
Missing	11 (0.1%)	6 (0.0%)	5 (0.1%)	
Household income (quartile)				<0.001
Lowest	3902 (14.5%)	2222 (12.4%)	1680 (18.8%)	
Lower-middle	4930 (22.5%)	3156 (21.5%)	1774 (24.4%)	
Upper-middle	5624 (29.9%)	3899 (31.2%)	1725 (27.2%)	
Highest	5997 (32.8%)	4198 (34.5%)	1799 (29.4%)	
Missing	51 (0.2%)	41 (0.3%)	10 (0.2%)	
Married	16,630 (74.3%)	11,241 (77.4%)	5389 (68.2%)	<0.001
Health-related behaviors				
Alcohol consumption				<0.001
≤1 time per month	10,129 (48.2%)	6519 (46.7%)	3610 (51.1%)	
2–4 times per month	4243 (22.9%)	3032 (24.2%)	1211 (20.3%)	
2–3 times per week	2714 (14.8%)	1962 (16.2%)	752 (11.9%)	
≥4 times per week	1136 (5.8%)	785 (6.1%)	351 (5.3%)	
Missing	2282 (8.3%)	1218 (6.8%)	1064 (11.4%)	
Smoking status				<0.001
Never smoker	12,645 (57.9%)	7850 (54.2%)	4795 (65.4%)	
Current smoker	3034 (17.3%)	2288 (19.5%)	746 (12.9%)	
Past smoker	4825 (24.8%)	3378 (26.4%)	1447 (21.8%)	
Dietary factors				
Breakfast frequency				0.004
5–7 times a week	12,559 (53.6%)	8084 (52.6%)	4475 (55.6%)	
3–4 times a week	1976 (11.0%)	1335 (11.0%)	641 (11.0%)	
1–2 times a week	2345 (13.9%)	1604 (14.4%)	741 (13.0%)	
0 times a week	3624 (21.4%)	2493 (22.0%)	1131 (20.3%)	
Total energy intake (kcal/day)	1856.09 (7.87)	1897.40 (8.56)	1773.40 (13.51)	<0.001
Carbohydrate intake (g/day)	258.90 (1.00)	263.80 (1.15)	249.10 (1.70)	<0.001
Protein intake (g/day)	71.38 (0.37)	72.71 (0.41)	68.72 (0.62)	<0.001
Fat intake (g/day)	50.59 (0.38)	51.97 (0.40)	47.83 (0.61)	<0.001
Sodium intake (mg/day)	3225.01 (16.93)	3303.68 (19.95)	3067.56 (27.60)	<0.001
Total dietary fiber intake (g/day)	24.60 (0.13)	24.73 (0.14)	24.33 (0.21)	<0.001
Total sugar intake (g/day)	57.47 (0.40)	59.56 (0.46)	53.29 (0.63)	<0.001
Comorbidities				
Hypertension, yes	5784 (22.8%)	3675 (22.2%)	2109 (24.0%)	0.008
Diabetes, yes	2528 (10.1%)	1648 (10.0%)	880 (10.1%)	0.903
Dyslipidemia, yes	5407 (21.8%)	3517 (21.8%)	1890 (21.8%)	0.982

Note: Values are presented as unweighted n (weighted %) or mean ± standard error (SE). *p* values were calculated using design-based Pearson’s chi-square tests for categorical variables and design-based Kruskal–Wallis tests for continuous variables, accounting for the complex sampling design. Abbreviations: KNHANES, Korea National Health and Nutrition Examination Survey; SE, standard error. “Non-consumers” refers to participants with 0 mg/day caffeine intake on the 24 h dietary recall day and should not be interpreted as habitual caffeine abstainers.

**Table 2:** Mental health- and sleep-related outcomes according to caffeine consumption.

Characteristic	Overall	0 mg/day	<100 mg/day	100 to <200 mg/day	200 to <300 mg/day	300 to <400 mg/day	≥400 mg/day	*p* Value
	N = 20,504 (100%)	N = 6988 (33.3%)	N = 4943 (22.6%)	N = 4812 (23.4%)	N = 1896 (10.1%)	N = 1163 (6.5%)	N = 702 (4.1%)	
GAD-7 score	2.18 ± 0.03	2.32 ± 0.05	1.97 ± 0.06	2.02 ± 0.05	2.19 ± 0.09	2.57 ± 0.12	2.48 ± 0.13	<0.001
GAD-7 score group								
0	10,309 (47.3%)	3474 (46.2%)	2655 (51.1%)	2466 (48.5%)	926 (46.0%)	506 (42.3%)	282 (40.0%)	
1 to <5	6917 (36.0%)	2334 (36.2%)	1570 (34.2%)	1650 (36.1%)	664 (37.1%)	425 (36.8%)	274 (40.1%)	
5 to <7	1279 (6.7%)	412 (6.3%)	278 (5.7%)	299 (6.7%)	129 (7.2%)	93 (9.4%)	68 (9.4%)	
7 to <10	1042 (5.3%)	378 (5.8%)	237 (4.8%)	229 (5.0%)	94 (5.2%)	66 (5.3%)	38 (5.0%)	
≥10	957 (4.7%)	390 (5.5%)	203 (4.1%)	168 (3.7%)	83 (4.4%)	73 (6.2%)	40 (5.4%)	
Average sleep duration (hours/day)	7.14 ± 0.01	7.18 ± 0.02	7.12 ± 0.02	7.13 ± 0.02	7.10 ± 0.03	7.15 ± 0.04	7.01 ± 0.05	0.002
Average sleep duration								<0.001
<6 h	2753 (12.5%)	1018 (13.2%)	626 (11.8%)	607 (11.9%)	239 (12.2%)	145 (12.3%)	118 (16.7%)	
6 to <7 h	4944 (24.7%)	1597 (23.4%)	1184 (24.8%)	1166 (25.0%)	507 (26.6%)	310 (27.4%)	180 (25.4%)	
7 to 8 h	9269 (45.4%)	3045 (43.7%)	2311 (47.1%)	2221 (46.3%)	881 (47.0%)	515 (44.2%)	296 (42.0%)	
>8 h	3538 (17.4%)	1328 (19.8%)	822 (16.4%)	818 (16.9%)	269 (14.2%)	193 (16.2%)	108 (15.8%)	
Perceived stress, yes	4983 (25.8%)	1698 (26.0%)	1086 (23.2%)	1126 (24.9%)	489 (27.7%)	339 (29.0%)	245 (34.7%)	<0.001

Note: Values are presented as unweighted n (weighted %) or mean ± standard error. *p* values were calculated using design-based Pearson’s chi-square test for categorical variables or design-based Kruskal–Wallis test for continuous variables, accounting for the complex sampling design. Abbreviations: GAD-7, Generalized Anxiety Disorder-7; KNHANES, Korea National Health and Nutrition Examination Survey; SE, standard error.

**Table 3:** Fully adjusted odds ratios (95% confidence intervals) for mental health outcomes according to caffeine intake categories in the overall population and stratified by sex and age group.

Outcome	0 mg/day	<100 mg/day	100 to <200 mg/day	200 to <300 mg/day	300 to <400 mg/day	≥400 mg/day
Overall (*n* = 20,504)						
GAD-7 score ≥ 10	1.29 (1.05–1.59)	1.00 (Reference)	0.94 (0.73–1.21)	1.05 (0.76–1.46)	1.39 (1.01–1.91)	1.25 (0.85–1.84)
Short sleep duration	1.24 (1.09–1.41)	1.00 (Reference)	1.08 (0.93–1.25)	1.19 (0.98–1.44)	1.33 (1.06–1.66)	1.85 (1.44–2.37)
Perceived stress	1.11 (1.00–1.24)	1.00 (Reference)	1.03 (0.93–1.15)	1.08 (0.93–1.24)	1.03 (0.87–1.21)	1.33 (1.10–1.61)
Male (*n* = 8848)						
GAD-7 score ≥ 10	1.61 (1.09–2.38)	1.00 (Reference)	1.13 (0.73–1.74)	0.92 (0.54–1.57)	1.70 (1.01–2.86)	1.25 (0.69–2.27)
Short sleep duration	1.25 (1.01–1.55)	1.00 (Reference)	0.95 (0.76–1.20)	1.13 (0.87–1.46)	1.20 (0.87–1.66)	1.73 (1.23–2.44)
Perceived stress	1.20 (1.01–1.44)	1.00 (Reference)	1.09 (0.91–1.30)	1.26 (1.02–1.55)	1.10 (0.86–1.40)	1.43 (1.10–1.86)
Female (*n* = 11,656)						
GAD-7 score ≥ 10	1.14 (0.90–1.45)	1.00 (Reference)	0.85 (0.63–1.14)	1.17 (0.78–1.75)	1.20 (0.80–1.79)	1.36 (0.82–2.25)
Short sleep duration	1.24 (1.07–1.44)	1.00 (Reference)	1.21 (1.00–1.45)	1.26 (0.96–1.67)	1.47 (1.08–2.00)	1.88 (1.28–2.75)
Perceived stress	1.04 (0.91–1.18)	1.00 (Reference)	1.00 (0.87–1.14)	0.90 (0.74–1.09)	0.96 (0.76–1.20)	1.25 (0.95–1.65)
Age < 65 (*n* = 14,201)						
GAD-7 score ≥ 10	1.25 (0.98–1.59)	1.00 (Reference)	0.87 (0.65–1.15)	1.00 (0.70–1.43)	1.34 (0.96–1.88)	1.10 (0.73–1.67)
Short sleep duration	1.16 (0.97–1.39)	1.00 (Reference)	1.08 (0.90–1.29)	1.27 (1.01–1.60)	1.24 (0.96–1.59)	1.81 (1.38–2.37)
Perceived stress	1.14 (1.00–1.29)	1.00 (Reference)	1.05 (0.93–1.19)	1.10 (0.94–1.29)	1.06 (0.88–1.26)	1.33 (1.08–1.63)
Age ≥ 65 (*n* = 6303)						
GAD-7 score ≥ 10	1.51 (1.05–2.16)	1.00 (Reference)	1.25 (0.80–1.97)	1.22 (0.61–2.43)	1.91 (0.68–5.37)	3.88 (1.22–12.39)
Short sleep duration	1.32 (1.11–1.58)	1.00 (Reference)	1.12 (0.89–1.41)	0.79 (0.55–1.13)	1.93 (1.09–3.41)	1.79 (0.98–3.26)
Perceived stress	1.10 (0.91–1.32)	1.00 (Reference)	0.91 (0.72–1.13)	0.87 (0.60–1.26)	1.00 (0.56–1.80)	1.72 (0.88–3.34)

Note: Odds ratios (ORs) and 95% confidence intervals (CIs) were estimated using survey-weighted generalized linear models with a quasi-binomial distribution. The <100 mg/day category was used as the reference group. Models were adjusted for sex (except in sex-stratified analyses), age group (except in age-stratified analyses), body mass index, education, household income, marital status, alcohol consumption, smoking status, breakfast frequency, total energy intake, carbohydrate, protein, fat, sodium, dietary fiber, total sugar intake, hypertension, diabetes mellitus, and dyslipidemia.

## Data Availability

The data presented in this study were derived from the Korea National Health and Nutrition Examination Survey (KNHANES) and are available from the Korea Disease Control and Prevention Agency (KDCA) at https://knhanes.kdca.go.kr (accessed on 28 July 2026).

## Supplementary Materials

The following supporting information can be downloaded at https://www.mdpi.com/article/10.3390/nu18152488/s1, Supplementary Table S1: Beverage classification criteria, assigned caffeine concentrations, and KNHANES dish codes used to estimate beverage-derived caffeine intake. Supplementary Table S2: Fully adjusted odds ratios (95% confidence intervals) for mental health outcomes according to caffeine intake categories in the primary and sensitivity analyses. Supplementary Table S3: Distribution of beverage-derived caffeine intake among Korean adults by sex and age, KNHANES 2021–2024. Supplementary Table S4: Predicted adjusted odds ratios (95% confidence intervals) at selected caffeine intake levels from the restricted cubic spline models shown in Figure 2. Supplementary Table S5: Associations between caffeine intake categories and mental health outcomes across sequential adjustment models.

## References

[1] Rauf M.Q., Sharma L., Essiet E., Elhassan O., Fahim R., Irem-Oko F., Sreedharan J. (2025). Caffeine Consumption Patterns, Health Impacts, and Media Influence: A Narrative Review. Cureus.

[2] Clark I., Landolt H.P. (2017). Coffee, Caffeine, and Sleep: A Systematic Review of Epidemiological Studies and Randomized Controlled Trials. Sleep Med. Rev..

[3] Hachenberger J., Li Y.-M., Realo A., Lemola S. (2025). The Association of Caffeine Consumption with Positive Affect but Not with Negative Affect Changes across the Day. Sci. Rep..

[4] Reichert C.F., Deboer T., Landolt H. (2022). Adenosine, Caffeine, and Sleep–Wake Regulation: State of the Science and Perspectives. J. Sleep Res..

[5] Gardiner C., Weakley J., Burke L.M., Roach G.D., Sargent C., Maniar N., Townshend A., Halson S.L. (2023). The Effect of Caffeine on Subsequent Sleep: A Systematic Review and Meta-Analysis. Sleep Med. Rev..

[6] Saarinen E.K., Kuusimäki T., Lindholm K., Niemi K., Honkanen E.A., Noponen T., Seppänen M., Ihalainen T., Murtomäki K., Mertsalmi T. (2024). Dietary Caffeine and Brain Dopaminergic Function in Parkinson Disease. Ann. Neurol..

[7] Kapellou A., King A., Graham C.A.M., Pilic L., Mavrommatis Y. (2023). Genetics of caffeine and brain-related outcomes—A systematic review of observational studies and randomized trials. Nutr. Rev..

[8] Chaudhary N.S., Grandner M.A., Jackson N.J., Chakravorty S. (2016). Caffeine Consumption, Insomnia, and Sleep Duration: Results from a Nationally Representative Sample. Nutrition.

[9] Distelberg B.J., Staack A., Elsen K.D., Sabaté J. (2017). The Effect of Coffee and Caffeine on Mood, Sleep, and Health-Related Quality of Life. J. Caffeine Res..

[10] Liu C., Wang L., Zhang C., Hu Z., Tang J., Xue J., Lu W. (2024). Caffeine Intake and Anxiety: A Meta-Analysis. Front. Psychol..

[11] Jagannath A., Varga N., Dallmann R., Rando G., Gosselin P., Ebrahimjee F., Taylor L., Mosneagu D., Stefaniak J., Walsh S. (2021). Adenosine Integrates Light and Sleep Signalling for the Regulation of Circadian Timing in Mice. Nat. Commun..

[12] Shah A.S., Pant M.R., Bommasamudram T., Nayak K.R., Roberts S.S.H., Gallagher C., Vaishali K., Edwards B.J., Tod D., Davis F. (2025). Effects of Sleep Deprivation on Physical and Mental Health Outcomes: An Umbrella Review. Am. J. Lifestyle Med..

[13] Fasokun M., Akinyemi O., Ogunyankin F., Ndebele-Ngwenya P., Gordon K., Ikugbayigbe S., Nwosu U., Michael M., Hughes K., Ogundare T. (2026). Associations between Sleep Duration and Depression, Mental Health, Physical Health, and General Health in U.S. Adults: A Population-Based Study. PLoS ONE.

[14] Nouri-Majd S., Salari-Moghaddam A., Keshteli A.H., Afshar H., Esmaillzadeh A., Adibi P. (2022). Coffee and caffeine intake in relation to symptoms of psychological disorders among adults. Public Health Nutr..

[15] Hwang B.I., Lee J.-Y., Lim H.J., Huh R., Ryu M., Jee S.H., Kimm H. (2025). Association between Caffeinated Beverages Consumption and Sleep Quality of Urban Workers. Korean J. Heal. Promot..

[16] Sulaiman N., Ali A., Zakaria M.K., Abu Shahim M.R., Jean S.W., Jalil A.M.M. (2024). Caffeine Consumption, Sleep Quality and Mental Health Outcomes Among Malaysian University Students. Natl. J. Community Med..

[17] Cho J.A., Kim S., Shin H., Kim H., Park E.-C. (2024). The Association between High-Caffeine Drink Consumption and Anxiety in Korean Adolescents. Nutrients.

[18] Paz-Graniel I., Kose J., Babio N., Hercberg S., Galan P., Touvier M., Salas-Salvadó J., Andreeva V.A. (2022). Caffeine Intake and Its Sex-Specific Association with General Anxiety: A Cross-Sectional Analysis among General Population Adults. Nutrients.

[19] Lee S.-H., Shin C., Kim H., Jeon S.W., Yoon H.-K., Ko Y.-H., Pae C.-U., Han C. (2022). Validation of the Korean Version of the Generalized Anxiety Disorder 7 Self-Rating Scale. Asia-Pac. Psychiatry Off. J. Pac. Rim Coll. Psychiatr..

[20] Ministry of Food and Drug Safety (2020). Keep Your Caffeine Intake to Less than 4 Cups of Coffee for Adults and 2 Cans of Energy Drinks for Adolescents.

[21] Korea Disease Control and Prevention Agency (2025). Korea National Health and Nutrition Examination Survey (KNHANES IX, 2022–2024): Raw Data User Guide.

[22] Spitzer R.L., Kroenke K., Williams J.B.W., Löwe B. (2006). A Brief Measure for Assessing Generalized Anxiety Disorder: The GAD-7. Arch. Intern. Med..

[23] Grandner M.A., Hale L., Moore M., Patel N.P. (2010). Mortality Associated with Short Sleep Duration: The Evidence, the Possible Mechanisms, and the Future. Sleep Med. Rev..

[24] Cheah Y.K., Azahadi M., Phang S.N., Abd Manaf N.H. (2020). Sociodemographic, Lifestyle, and Health Factors Associated With Depression and Generalized Anxiety Disorder Among Malaysian Adults. J. Prim. Care Community Health.

[25] Huang Y., Loux T. (2023). A Cross-Sectional Study: Association between Tobacco/Alcohol Usage and Mental Health with Disabilities. Ment. Health Prev..

[26] Cifuentes L., Campos A., Silgado M.L.R., Kelpin S., Stutzman J., Hurtado M.D., Grothe K., Hensrud D.D., Clark M.M., Acosta A. (2022). Association between Anxiety and Eating Behaviors in Patients with Obesity. Obes. Pillars.

[27] Chen Y., Wu C., Qian W. (2025). Underestimated Anxiety in Chronic Diseases: A Cross-Sectional Study on Specific Risk Factors. Medicine.

[28] Xue Y., Wang W.-D., Liu Y.-J., Wang J., Walters A.S. (2025). Sleep Disturbances in Generalized Anxiety Disorder: The Central Role of Insomnia. Sleep Med..

[29] Unsal S., Sanlier N. (2025). Longitudinal Effects of Lifetime Caffeine Consumption on Levels of Depression, Anxiety, and Stress: A Comprehensive Review. Curr. Nutr. Rep..

[30] Rodak K., Kokot I., Kratz E.M. (2021). Caffeine as a Factor Influencing the Functioning of the Human Body—Friend or Foe?. Nutrients.

[31] Lee J., Park H., Han S., Tana G., Chang M. (2019). Study on relationship between caffeine intake level and metabolic syndrome and related diseases in Korean adults: 2013~2016 Korea National Health and Nutrition Examination Survey. J. Nutr. Health.

[32] Institute of Medicine (2014). Caffeine Effects on the Central Nervous System and Behavioral Effects Associated with Caffeine Consumption. Caffeine in Food and Dietary Supplements: Examining Safety: Workshop Summary.

[33] do Nascimento A.O., da Silva C.E.C., da Silva M.L., Cunha K.d.C. (2026). The Effects of Caffeine on Anxiety Behavior in Healthy Individuals: A Systematic Review of the Literature. Stress Health.

[34] Walsh R.F.L., Smith L.T., Bisgay A., Stephenson A.R., Goel N., Alloy L.B. (2025). Sleep Duration as a Mediator of the Association between Caffeine Intake and Mood Symptoms: An Intensive Longitudinal Study of Young Adults with and without Bipolar Spectrum Disorders. Chronobiol. Int..

