# Supplementary material for: Beverage-Derived Caffeine Intake, Sleep Duration, and Mental Health Among Korean Adults: A Cross-Sectional Analysis of KNHANES 2021–2024
Source: Nutrients. 2026 Aug 1;18(15):2488. doi: 10.3390/nu18152488 (PMC13468444; doi:10.3390/nu18152488)
Supplement: Supplementary file 1 [file nutrients-18-02488-s001.zip › Supplementary Table S1.pdf]

Supplementary Table S1. Beverage classification criteria, assigned caffeine concentrations, and KNHANES dish codes used to estimate beverage-derived caffeine intake

Beverage classification	Assigned caffeine concentration (mg/L)	KNHANES dish codes (No. of codes; N_DCODE)	Records, n (%) (N=22,599)	Consumers, n (%) (N=13,516)	Representative examples
Coffee – Prepared	571.9	**14:** 2014, 2019, 2032, 2033, 2034, 2035, 2082, 2090, 2091, 2092, 2094, 2095, 2099, 5016	9,172 (40.6%)	5,823 (43.1%)	**Examples:** Coffee mix, vending coffee, instant latte.<br>**Include:** 커피, 믹스, 라떼, 모카, 카누, 맥심.<br>**Exclude:** 녹차, 홍차, 코코아, 주스, 허브티.<br>**Decaffeinated:** 디카페인
Coffee – Roasted	522.8	**12:** 2032, 2033, 2034, 2035, 2044, 2090, 2091, 2094, 2095, 2099, 5003, 5016	6,268 (27.7%)	4,712 (34.9%)	**Examples:** Americano, espresso, drip coffee.<br>**Include:** 아메리카노, 에스프레소, 원두, 드립, 캡슐.<br>**Exclude:** 녹차, 홍차, 코코아.<br>**Decaffeinated:** 디카페인
Coffee – Ready-to-drink	352.9	**10:** 2032, 2033, 2034, 2035, 2044, 2081, 2090, 2091, 2094, 5016	721 (3.2%)	557 (4.1%)	**Examples:** Georgia, Cantata, Let's Be.<br>**Include:** 캔커피, 병커피, 조지아, 칸타타, 레쓰비.<br>**Exclude:** 녹차, 홍차, 코코아.<br>**Decaffeinated:** 디카페인
Coffee – Instant powder	293.5	**12:** 2028, 2032, 2033, 2034, 2035, 2044, 2081, 2090, 2094, 2095, 2099, 5016	3,919 (17.3%)	2,789 (20.6%)	**Examples:** Instant coffee powder, G7, Maxim Black.<br>**Include:** 가루, 분말, 인스턴트, 스틱.<br>**Exclude:** 녹차, 홍차, 코코아.<br>**Decaffeinated:** 디카페인
Cola	187.5	**2:** 2065, 5013	1,598 (7.1%)	1,422 (10.5%)	**Examples:** Coca-Cola, Pepsi.<br>**Include:** 콜라, 펩시.<br>**Exclude:** 맥콜, 콜라겐, 주스.
Tea – Infusion	143.6	**8:** 2002, 2011, 2025, 2030, 2044, 2064, 2069, 2090	417 (1.8%)	335 (2.5%)	**Examples:** Green tea, black tea, oolong tea.<br>**Include:** 녹차, 홍차, 우롱차, 말차.<br>**Exclude:** 율무차, W 차, 허브티.

Beverage classification	Assigned caffeine concentration (mg/L)	KNHANES dish codes (No. of codes; N_DCODE)	Records, n (%) (N=22,599)	Consumers, n (%) (N=13,516)	Representative examples
Tea – Powder	115	**7:** 2002, 2014, 2019, 2026, 2044, 5011, 5012	137 (0.6%)	129 (1.0%)	**Examples:** Matcha powder, tea powder.<br>**Include:** 녹차, 말차, 홍차.<br>**Exclude:** 율무차, W 차.<br>**Minimal-caffeine:** 아이스티
Tea – Ready-to-drink	105.8	**11:** 2002, 2010, 2014, 2026, 2044, 2068, 2090, 2094, 5011, 5013, 5020	75 (0.3%)	69 (0.5%)	**Examples:** Bottled green tea.<br>**Include:** 녹차, 홍차, 우롱차.<br>**Exclude:** 율무차, W 차.<br>**Minimal-caffeine:** 아이스티
Energy drink	320.8	**8:** 2002, 2039, 2044, 2058, 2065, 2066, 5013, 5023	260 (1.2%)	236 (1.7%)	**Examples:** Hot6, Monster, Red Bull, Bacchus.<br>**Include:** 핫식스, 몬스터, 레드불, 박카스.<br>**Exclude:** 커피, 코코아.
Cocoa	360.9	**4:** 2027, 2037, 2083, 2090	32 (0.1%)	27 (0.2%)	**Examples:** Hot cocoa, chocolate drink.<br>**Include:** 코코아, 초코, 카카오.<br>**Exclude:** 커피, 모카.<br>**Decaffeinated:** 카카오닙스차

Table footnotes:

Example items are representative examples and do not constitute the complete list of products.

Keywords are presented in Korean because KNHANES dietary records are recorded in Korean and beverage classification was performed using Korean text matching.

Beverage classification was performed using predefined Korean keyword matching and KNHANES dish codes, followed by manual review of ambiguous records before assigning beverage-specific caffeine concentrations.

Assigned caffeine concentrations were based on the mean caffeine concentrations reported in the Korean Ministry of Food and Drug Safety (MFDS) caffeine content database.

KNHANES dish codes (N_DCODE) are five-digit standardized identifiers used in the KNHANES dietary database. Beverage records were identified using the last four digits of the dish code and Korean food names, followed by manual review to ensure mutually exclusive beverage classifications.

Decaffeinated and minimal-caffeine keywords indicate text-matching terms used to identify beverage records for which a minimal caffeine concentration (1 mg/L) was assigned. These terms were used for analytical classification purposes only and do not necessarily indicate products formally labeled as decaffeinated or caffeine-free.

A participant could contribute multiple beverage records because each beverage item reported in the KNHANES 24-hour dietary recall was recorded separately.
Record-level percentages were calculated using the 22,599 mutually exclusive beverage records contributed by the 13,516 participants with beverage-derived caffeine intake >0 mg/day. Consumer-level percentages were calculated using 13,516 unique participants as the denominator. Because participants could consume beverages from multiple categories, consumer percentages may sum to more than 100%.

Korean keyword definitions: 커피, coffee; 믹스, coffee mix; 라떼, latte; 모카, mocha; 카누, KANU; 맥심, Maxim; 녹차, green tea; 홍차, black tea; 코코아, cocoa; 주스, juice; 허브티, herbal tea; 디카페인, decaffeinated; 아메리카노, Americano; 에스프레소, espresso; 원두, coffee beans; 드립, drip coffee; 캡슐, coffee capsule; 캔커피, canned coffee; 병커피, bottled coffee; 조지아, Georgia; 칸타타, Cantata; 레쓰비, Let's Be; 가루/분말, powder; 인스턴트, instant; 스틱, stick-type product; 콜라, cola; 펩시, Pepsi; 맥콜, McCol; 콜라겐, collagen; 우롱차, oolong tea; 말차, matcha; 율무차, Job's tears tea; W 차, W Tea; 아이스티, iced tea; 핫식스, HOT6; 몬스터, Monster; 레드불, Red Bull; 박카스, Bacchus; 초코, chocolate; 카카오, cacao; 카카오닙스차, cacao nib tea.
