# Supplementary material for: Beverage-Derived Caffeine Intake, Sleep Duration, and Mental Health Among Korean Adults: A Cross-Sectional Analysis of KNHANES 2021–2024
Source: Nutrients. 2026 Aug 1;18(15):2488. doi: 10.3390/nu18152488 (PMC13468444; doi:10.3390/nu18152488)
Supplement: Supplementary file 1 [file nutrients-18-02488-s001.zip › Supplementary Table S2.pdf]

Supplementary Table S2. Sensitivity analyses: Fully adjusted odds ratios (95% confidence intervals) for mental health outcomes according to caffeine intake categories

Outcome	0 mg/day	<100 mg/day	100 to <200 mg/day	200 to <300 mg/day	300 to <400 mg/day	≥400 mg/day
**Primary analysis (n = 20,504)**						
Clinically significant anxiety	1.29 (1.05–1.59)	1.00 (Reference)	0.94 (0.73–1.21)	1.05 (0.76–1.46)	1.39 (1.01–1.91)	1.25 (0.85–1.84)
Short sleep duration	1.24 (1.09–1.41)	1.00 (Reference)	1.08 (0.93–1.25)	1.19 (0.98–1.44)	1.33 (1.06–1.66)	1.85 (1.44–2.37)
Perceived stress	1.11 (1.00–1.24)	1.00 (Reference)	1.03 (0.93–1.15)	1.08 (0.93–1.24)	1.03 (0.87–1.21)	1.33 (1.10–1.61)
**Sensitivity I. Assigning a caffeine concentration of 0 mg/L to cocoa beverages (n = 20,504)**						
Clinically significant anxiety	1.28 (1.04–1.58)	1.00 (Reference)	0.94 (0.73–1.20)	1.05 (0.76–1.46)	1.39 (1.01–1.91)	1.25 (0.84–1.84)
Short sleep duration	1.24 (1.09–1.41)	1.00 (Reference)	1.08 (0.93–1.25)	1.19 (0.99–1.45)	1.33 (1.06–1.66)	1.85 (1.44–2.37)
Perceived stress	1.11 (1.00–1.24)	1.00 (Reference)	1.03 (0.93–1.15)	1.08 (0.94–1.25)	1.03 (0.87–1.21)	1.33 (1.10–1.62)
**Sensitivity II. Assigning a caffeine concentration of 0 mg/L to cocoa and decaffeinated beverages (n = 20,504)**						
Clinically significant anxiety	1.25 (1.01–1.54)	1.00 (Reference)	0.93 (0.72–1.19)	1.04 (0.75–1.44)	1.38 (1.00–1.89)	1.23 (0.83–1.82)
Short sleep duration	1.25 (1.10–1.42)	1.00 (Reference)	1.09 (0.94–1.26)	1.21 (1.00–1.46)	1.35 (1.08–1.68)	1.87 (1.45–2.39)
Perceived stress	1.14 (1.02–1.27)	1.00 (Reference)	1.05 (0.94–1.17)	1.10 (0.95–1.27)	1.05 (0.88–1.24)	1.36 (1.12–1.65)
**Sensitivity III. Excluding pregnant women and participants with implausible energy intake (n = 20,199)**						
Clinically significant anxiety	1.23 (0.99–1.53)	1.00 (Reference)	0.92 (0.71–1.19)	1.06 (0.76–1.47)	1.37 (0.99–1.90)	1.18 (0.78–1.78)
Short sleep duration	1.24 (1.09–1.40)	1.00 (Reference)	1.09 (0.94–1.26)	1.22 (1.01–1.48)	1.31 (1.04–1.64)	1.89 (1.47–2.43)
Perceived stress	1.15 (1.03–1.28)	1.00 (Reference)	1.05 (0.94–1.17)	1.10 (0.95–1.27)	1.02 (0.86–1.21)	1.35 (1.11–1.64)
**Sensitivity IV. Excluding dietary covariates from the fully adjusted model (n = 20,199)**						
Clinically significant anxiety	1.21 (0.98–1.50)	1.00 (Reference)	0.92 (0.71–1.19)	1.07 (0.77–1.49)	1.39 (1.00–1.92)	1.19 (0.79–1.79)
Short sleep duration	1.23 (1.08–1.39)	1.00 (Reference)	1.09 (0.94–1.26)	1.23 (1.02–1.49)	1.32 (1.05–1.65)	1.90 (1.48–2.43)
Perceived stress	1.14 (1.02–1.27)	1.00 (Reference)	1.05 (0.94–1.17)	1.11 (0.96–1.29)	1.04 (0.88–1.23)	1.38 (1.14–1.68)
**Sensitivity V. Complete-case analysis excluding participants with missing values in any covariates (n = 17,684)**						
Clinically significant anxiety	1.21 (0.96–1.53)	1.00 (Reference)	0.94 (0.72–1.24)	1.13 (0.81–1.60)	1.42 (1.02–1.99)	1.12 (0.73–1.72)
Short sleep duration	1.23 (1.07–1.42)	1.00 (Reference)	1.09 (0.93–1.27)	1.22 (0.99–1.49)	1.34 (1.06–1.69)	1.90 (1.46–2.46)
Perceived stress	1.14 (1.01–1.28)	1.00 (Reference)	1.06 (0.94–1.19)	1.10 (0.95–1.29)	1.04 (0.87–1.23)	1.33 (1.09–1.63)

Table footnotes:

Values are fully adjusted odds ratios (ORs) with 95% confidence intervals (CIs). The <100 mg/day category was used as the reference group. The fully adjusted model included sex, age group, body mass index, education, household income, marital status, alcohol consumption, smoking status, breakfast frequency, total energy intake, carbohydrate, protein, fat, sodium, dietary fiber, total sugar intake, hypertension, diabetes mellitus, and dyslipidemia.

Sensitivity analyses were performed cumulatively. Sensitivity analysis I assigned a caffeine concentration of 0 mg/L to cocoa beverages. Sensitivity analysis II additionally assigned a caffeine concentration of 0 mg/L to decaffeinated beverages. Sensitivity analysis III additionally excluded pregnant women and participants with implausible total energy intake (<500 or ≥5000 kcal/day). Sensitivity analysis IV additionally excluded dietary covariates (breakfast frequency, total energy intake, carbohydrate, protein, fat, sodium, dietary fiber, and total sugar intake) from the fully adjusted model. Sensitivity analysis V additionally excluded participants with missing values for any covariates included in the fully adjusted model (complete-case analysis).
