# Supplementary material for: Beverage-Derived Caffeine Intake, Sleep Duration, and Mental Health Among Korean Adults: A Cross-Sectional Analysis of KNHANES 2021–2024
Source: Nutrients. 2026 Aug 1;18(15):2488. doi: 10.3390/nu18152488 (PMC13468444; doi:10.3390/nu18152488)
Supplement: Supplementary file 1 [file nutrients-18-02488-s001.zip › Supplementary Table S3.pdf]

Supplementary Table S3. Distribution of beverage-derived caffeine intake among Korean adults by sex and age, KNHANES 2021–2024.

Subgroup	n	Weighted population	Mean ± SE (mg/day)	P25	P50 (Median)	P75	P95	P99	Maximum
**Overall**	20,504	42,684,846	117.5 ± 1.3	0	75.8	185.6	376.4	622.1	4,735
**Male**	8,848	21,273,021	132.3 ± 2.0	0	88.1	196.2	411.8	667.8	4,735
**Female**	11,656	21,411,825	102.9 ± 1.6	0	68.6	168.7	343.1	555.1	1,882
**Age <65 years**	14,201	33,637,520	129.1 ± 1.6	0	86.2	188.2	393.1	633.3	4,735
**Age ≥65 years**	6,303	9,047,325	74.6 ± 1.4	0	57.2	114.4	245.4	411.8	1,701

Table footnote:

Values are presented as weighted estimates accounting for the complex sampling design of KNHANES. Percentiles were calculated from the weighted distribution of beverage-derived caffeine intake. The overall distribution includes both caffeine consumers and non-consumers.

The reference value of 100 mg/day used in the restricted cubic spline analyses corresponds to a common level of caffeine intake in the study population and is consistent with the reference category (100–<200 mg/day) used in the categorical analyses.
