# Supplementary material for: Beverage-Derived Caffeine Intake, Sleep Duration, and Mental Health Among Korean Adults: A Cross-Sectional Analysis of KNHANES 2021–2024
Source: Nutrients. 2026 Aug 1;18(15):2488. doi: 10.3390/nu18152488 (PMC13468444; doi:10.3390/nu18152488)
Supplement: Supplementary file 1 [file nutrients-18-02488-s001.zip › Supplementary Table S4.pdf]

Supplementary Table S4. Predicted adjusted odds ratios (95% confidence intervals) at selected caffeine intake levels from restricted cubic spline models shown in Figure 2

Outcome (n = 20,504)	Caffeine intake (mg/day) 0	100	200	300	400	500	600	P-overall	P-nonlinear
**Crude model**									
Clinically significant anxiety	1.48 (1.25–1.76)	1.00 (Reference)	1.17 (1.00–1.37)	1.27 (1.04–1.56)	1.35 (1.09–1.66)	1.42 (1.13–1.78)	1.50 (1.16–1.93)	<0.001	<0.001
Short sleep duration	1.13 (1.01–1.27)	1.00 (Reference)	0.99 (0.90–1.08)	1.06 (0.95–1.19)	1.20 (1.06–1.35)	1.35 (1.16–1.56)	1.52 (1.25–1.83)	<0.001	0.263
Perceived stress	1.19 (1.08–1.30)	1.00 (Reference)	1.21 (1.12–1.30)	1.34 (1.23–1.47)	1.44 (1.29–1.60)	1.53 (1.34–1.76)	1.64 (1.37–1.96)	<0.001	<0.001
**Fully adjusted model**									
Clinically significant anxiety	1.33 (1.11–1.59)	1.00 (Reference)	1.11 (0.94–1.31)	1.18 (0.96–1.45)	1.23 (0.99–1.52)	1.28 (1.02–1.62)	1.34 (1.03–1.75)	0.010	0.016
Short sleep duration	1.24 (1.10–1.39)	1.00 (Reference)	1.13 (1.03–1.25)	1.28 (1.14–1.44)	1.45 (1.27–1.64)	1.63 (1.40–1.90)	1.84 (1.51–2.23)	<0.001	<0.001
Perceived stress	1.13 (1.02–1.24)	1.00 (Reference)	1.05 (0.97–1.13)	1.09 (0.99–1.20)	1.14 (1.02–1.27)	1.19 (1.04–1.35)	1.23 (1.05–1.46)	0.025	0.062

Table footnotes:

Predicted odds ratios (ORs) and 95% confidence intervals (CIs) were estimated from the crude and fully adjusted restricted cubic spline models, with 100 mg/day specified as the reference value.

The fully adjusted model included sex, age group, body mass index, education, household income, marital status, alcohol consumption, smoking status, breakfast frequency, total energy intake, carbohydrate, protein, fat, sodium, dietary fiber, total sugar intake, hypertension, diabetes mellitus, and dyslipidemia.

Predicted ORs were calculated at selected caffeine intake levels (0, 100, 200, 300, 400, 500, and 600 mg/day) to facilitate interpretation of the restricted cubic spline analyses shown in Figure 2.

P-overall and P-nonlinear were obtained using Wald tests for the overall association and nonlinear component of the restricted cubic spline models, respectively.
