# Supplementary material for: Beverage-Derived Caffeine Intake, Sleep Duration, and Mental Health Among Korean Adults: A Cross-Sectional Analysis of KNHANES 2021–2024
Source: Nutrients. 2026 Aug 1;18(15):2488. doi: 10.3390/nu18152488 (PMC13468444; doi:10.3390/nu18152488)
Supplement: Supplementary file 1 [file nutrients-18-02488-s001.zip › Supplementary Table S5.pdf]

Supplementary Table S5. Odds ratios (95% confidence intervals) for mental health outcomes according to caffeine intake categories across sequential adjustment models

	Model 0<br>OR (95% CI)	Model 1<br>OR (95% CI)	Model 2<br>OR (95% CI)	Model 3<br>OR (95% CI)	Model 4<br>OR (95% CI)	Model 5<br>OR (95% CI)
**GAD-7 score ≥10**						
Non-consumers	**1.38 (1.13–1.68)**	**1.28 (1.04–1.56)**	**1.26 (1.03–1.54)**	**1.26 (1.03–1.55)**	**1.29 (1.05–1.58)**	**1.29 (1.05–1.59)**
<100mg	Reference	Reference	Reference	Reference	Reference	Reference
100 to <200mg	0.90 (0.71–1.15)	0.90 (0.70–1.15)	0.95 (0.74–1.22)	0.93 (0.72–1.19)	0.93 (0.72–1.20)	0.94 (0.73–1.21)
200 to <300mg	1.07 (0.78–1.47)	1.07 (0.77–1.46)	1.14 (0.83–1.56)	1.06 (0.77–1.47)	1.05 (0.76–1.45)	1.05 (0.76–1.46)
300 to <400mg	**1.55 (1.14–2.09)**	**1.41 (1.03–1.92)**	**1.53 (1.12–2.08)**	**1.41 (1.03–1.93)**	**1.39 (1.01–1.90)**	**1.39 (1.01–1.91)**
≥400mg	1.34 (0.92–1.95)	1.35 (0.92–1.97)	1.42 (0.97–2.09)	1.28 (0.87–1.88)	1.26 (0.85–1.86)	1.25 (0.85–1.84)
**Sleep duration < 6h**						
Non-consumers	**1.14 (1.00–1.29)**	**1.22 (1.07–1.38)**	**1.23 (1.08–1.39)**	**1.23 (1.08–1.39)**	**1.24 (1.09–1.41)**	**1.24 (1.09–1.41)**
<100mg	Reference	Reference	Reference	Reference	Reference	Reference
100 to <200mg	1.01 (0.88–1.17)	1.06 (0.92–1.22)	1.09 (0.94–1.26)	1.08 (0.93–1.25)	1.08 (0.93–1.24)	1.08 (0.93–1.25)
200 to <300mg	1.04 (0.87–1.25)	1.19 (0.99–1.43)	1.22 (1.01–1.48)	1.20 (0.99–1.45)	1.19 (0.98–1.44)	1.19 (0.98–1.44)
300 to <400mg	1.05 (0.84–1.30)	1.30 (1.04–1.63)	1.36 (1.09–1.71)	1.33 (1.06–1.67)	1.32 (1.05–1.65)	1.33 (1.06–1.66)
≥400mg	**1.51 (1.18–1.92)**	**1.86 (1.45–2.39)**	**1.94 (1.51–2.49)**	**1.85 (1.44–2.37)**	**1.84 (1.43–2.36)**	**1.85 (1.44–2.37)**
**Perceived stress, yes**						
Non-consumers	1.16 (1.05–1.29)	1.08 (0.97–1.21)	1.08 (0.97–1.21)	1.10 (0.98–1.22)	1.11 (0.99–1.23)	1.11 (1.00–1.24)
<100mg	Reference	Reference	Reference	Reference	Reference	Reference
100 to <200mg	1.10 (0.99–1.22)	1.05 (0.94–1.16)	1.05 (0.95–1.17)	1.03 (0.92–1.14)	1.02 (0.92–1.14)	1.03 (0.93–1.15)
200 to <300mg	1.27 (1.10–1.46)	1.15 (0.99–1.32)	1.15 (1.00–1.33)	1.08 (0.94–1.25)	1.07 (0.93–1.24)	1.08 (0.93–1.24)
300 to <400mg	1.35 (1.15–1.58)	1.10 (0.94–1.30)	1.11 (0.94–1.31)	1.04 (0.88–1.22)	1.02 (0.87–1.21)	1.03 (0.87–1.21)
≥400mg	**1.76 (1.46–2.11)**	**1.50 (1.24–1.81)**	**1.51 (1.25–1.83)**	**1.37 (1.13–1.66)**	**1.34 (1.10–1.62)**	**1.33 (1.10–1.61)**

Table footnotes:

Model 0 was an unadjusted crude model.

Model 1 was adjusted for demographic and anthropometric factors, including sex, age group, and body mass index (BMI).

Model 2 additionally adjusted for socioeconomic status, including education level, household income quartile, and marital status.

Model 3 further included health-related behaviors, including alcohol consumption and smoking status.

Model 4 additionally adjusted for dietary factors, including breakfast frequency, total energy intake, and macronutrient/micronutrient intakes (carbohydrates, protein, fat, sodium, dietary fiber, and total sugars).

Model 5 further adjusted for chronic comorbidities, including hypertension, diabetes mellitus, and dyslipidemia.

All models were estimated using survey-weighted logistic regression accounting for the complex sampling design of the Korea National Health and Nutrition Examination Survey (KNHANES).

Abbreviations: CI, confidence interval; GAD-7, Generalized Anxiety Disorder-7; OR, odds ratio.
